# Human umbilical cord blood infusions in management of autism spectrum disorder: Narrative review

**DOI:** 10.1192/j.eurpsy.2021.1109

**Published:** 2021-08-13

**Authors:** M. Adnan, F. Motiwala, Z. Mansuri, A. Reddy

**Affiliations:** 1 Psychiatry, Mercy Hospital and Medical Center, Lincolnwood, United States of America; 2 Psychiatry, Texas Tech University Health Sciences Center at the Permian Basin, Midland, United States of America; 3 Department Of Psychiatry, Boston Children’s Hospital/Harvard Medical School, Boston, United States of America; 4 Psychiatry, Virginia Tech Carilion School of Medicine, Roanoke, United States of America

**Keywords:** autism spectrum disorder

## Abstract

**Introduction:**

According to CDC’s Autism and Developmental Disabilities Monitoring Network surveillance in 2016, autism spectrum disorder (ASD) was prevalent in 1 in 54 children in 11 states of the US.

**Objectives:**

This systematic review provides an overview of Umbilical Cord Blood Infusion (UCB) to decrease symptoms severity in children with (ASD).

**Methods:**

Systematic literature search was conducted using “Autism” OR “Autism spectrum disorder” AND “Autologous Umbilical Cord Blood Infusion (AUCBI)” OR “umbilical cord blood” OR “Allogeneic Cord Blood” in PubMed, Embase, and PsycINFO. Three studies were qualified on AUCBI.

**Results:**

We found 3 studies on UCBI The UCB Infusion phase-I/ open-label trial showed significant improvement in cognitive and behavior scales, especially in the social domain in the first six months, and was more significant in children with higher baseline nonverbal intelligence quotients. Other study/phase II trial failed to show any effects of UCBI on social communication, vocabulary, and other autism symptoms. On subgroup analysis, the improvement in Clinical Global Impression - Improvement (CGI-I) in children without intellectual disability (ID) with the allogenic (not autologous) UCBI was observed. Another randomized, blinded crossover trial failed to show any difference between improvements in CGI baseline severity scores in placebo vs. cord blood infusion groups.

**Conclusions:**

The data provides evidence to support the efficacy and safety of autologous UCBI in symptom severity reductions and improved clinical outcomes without intellectual disability. However, the evidence is inadequate and future large scale clinical are required.

